# Mortality trends and disparities for coexisting chronic obstructive pulmonary disease and cardiovascular disease: A retrospective analysis of deaths in the United States from 1999–2020

**DOI:** 10.1371/journal.pone.0317592

**Published:** 2025-02-04

**Authors:** Aman Goyal, Humza Saeed, Wania Sultan, Ajeet Singh, Abdullah, Muhammad Khubaib Arshad, Zubair Amin, Mah I Kan Changez, Gauranga Mahalwar, Rozi Khan, Wael AlJaroudi

**Affiliations:** 1 Department of Internal Medicine, Seth GS Medical College and KEM Hospital, Mumbai, India; 2 Department of Critical Care Medicine, Alchemist Hospitals, Panchkula, India,; 3 Department of Internal Medicine, Rawalpindi Medical University, Rawalpindi, Pakistan; 4 Department of Internal Medicine, Dow University of Health Sciences, Karachi, Pakistan; 5 Department of Cardiothoracic Surgery, Yale University, New Haven, Connecticut, United States of America; 6 Department of Internal Medicine, Cleveland Clinic Foundation, Cleveland, Ohio, United States of America; 7 Department of Internal Medicine, Medical University of South Carolina, Florence, South Carolina, United States of America; 8 Department of Cardiology, WellStar MCG Health, Augusta, Georgia, United States of America; La Trobe University - Bundoora Campus: La Trobe University, AUSTRALIA

## Abstract

**Background:**

Chronic obstructive pulmonary disease (COPD) and cardiovascular disease (CVD) greatly influence morbidity and mortality, with COPD patients frequently suffering from cardiovascular comorbidities like coronary heart disease and stroke. This study analyzes mortality trends and disparities among individuals in the United States (US) affected by both CVD and COPD.

**Methods:**

This study analyzed death certificates from the CDC WONDER database for individuals aged 25 and older who died between 1999 and 2020 with both CVD (ICD I00-I99) and COPD (ICD J41-J44). Age-adjusted mortality rates (AAMRs) and annual percent change (APC) were calculated by year, sex, age group, race/ethnicity, geographic region, and urbanization status.

**Results:**

Between 1999 and 2020, there were 3,590,124 reported deaths due to coexisting CVD and COPD, with overall AAMR slightly changing from 82.2 to 81.2 per 100,000 population, and a notable rise from 2018 to 2020 (APC: 5.28; 95% CI: 1.83 to 7.22) coinciding with the onset of COVID-19 pandemic. A similar surge in mortality was observed across multiple demographic subgroups, particularly among older adults. Disparities across age groups, sex, race, and geographic location were also observed in the mortality rates due to CVD and COPD. When analyzed by age group, older adults exhibited the highest AAMR at 824.1. Men had higher AAMRs than women (96.5 vs. 60.7). Ethnoracial analysis showed that non-Hispanic (NH) White individuals had the highest AAMRs (82.0), followed by NH American Indian or Alaska Native (74.5), NH Black (63.6), Hispanic (38.1), and NH Asian or Pacific Islander (25.1) individuals. Additionally, non-metropolitan areas had higher AAMRs compared to metropolitan areas (96.2 vs. 70.9).

**Conclusions:**

The findings suggest that mortality rates for CVD and COPD have increased in recent years, coinciding with the onset of the COVID-19 pandemic, which may have exacerbated outcomes in vulnerable populations. The study highlights the need for targeted interventions to address the overlapping impacts of CVD and COPD, especially in high-risk groups.

## Introduction

Chronic obstructive pulmonary disease (COPD) is a prevalent lung condition that encompasses emphysema and chronic bronchitis [1]. In 2021, COPD accounted for 138,825 deaths, ranking as the sixth overall cause of mortality and the fifth among disease-related deaths, following heart disease, cancer, COVID-19, accidents, and stroke in the United States (US) [2]. Notably, COPD disproportionately affects older age groups, particularly in low- and middle-income countries, ranking as the seventh leading cause of poor health globally [2].

Cardiovascular diseases (CVDs) play a pivotal role in the morbidity and mortality of mild-to-moderate COPD. Hospital-diagnosed COPD is associated with increased age-adjusted in-hospital mortality rates for pneumonia, hypertension, and heart failure [3]. The interplay between COPD and CVDs is evident, with COPD patients frequently experiencing comorbidities such as coronary heart disease (CHD), myocardial infarction (MI), hypertension, diabetes, and stroke [4,5]. Moreover, COPD patients face a 2–5 times higher risk of developing ischemic heart disease, cardiac dysrhythmia, heart failure, pulmonary circulation diseases, and arterial conditions compared to non-COPD patients [5].

Both CVDs and COPD share common risk factors, including cigarette smoking and sedentary lifestyles, as well as exhibit overlapping pathophysiologies such as low-grade pulmonary and systemic inflammation, lung hyperinflation, hypoxemia, pulmonary hypertension, and arterial stiffness [3,6]. Recent studies have highlighted that reduced pulmonary function is linked to an increased risk of developing CVD, with COPD patients more likely to experience cardiac diseases such as ischemic heart disease, myocardial infarction, and stroke [7–12]. This complex relationship is thought to be driven by shared risk factors and systemic inflammation, which contribute to the progression of both conditions [7,8,13]. Furthermore, COPD patients with cardiovascular complications experience longer hospital stays, more frequent hospitalizations, and higher mortality rates due to the combined effects of both diseases [5,14]. Recognizing this intricate association is crucial because treatments for COPD and CVD can significantly impact each other, potentially exacerbating one condition while managing the other [15].

Although health agencies like the CDC and WHO monitor CVD and COPD-related mortality separately [16,17], there is limited research on their combined impact on mortality, especially in populations with both conditions. Mortality rates serve as a crucial measure of disease burden, capturing both acute and chronic outcomes, and helping identify trends and disparities. While risk factors such as smoking, obesity, and diabetes are well-documented, their joint effect on CVD and COPD mortality has not been sufficiently explored. Additionally, the COVID-19 pandemic has likely influenced these trends, particularly for vulnerable groups. This study aims to address this gap by analyzing the combined effects of CVD and COPD on mortality, identifying patterns across demographic and geographic groups, and contributing to targeted prevention and intervention strategies.

## Methods

This study was a retrospective analysis aimed at determining trends in mortality and disparities in mortality rates due to CVD and COPD, with a focus on variations by sex, ethnoracial groups, age, and geographical variables.

### Study setting and population

We sourced the mortality data on June 20, 2024, from the Centers for Disease Control and Prevention’s WONDER (Wide-Ranging Online Data for Epidemiologic Research) database to investigate mortality rates related to CVD and COPD between 1999 and 2020 [18]. The Multiple Cause-of-Death Public Use record death certificates were studied to identify cases where both CVD (or any of their complications) and COPD were listed as either contributing or underlying cause of death on death certificates across the nation [19]. Patients with both CVD and COPD were identified using the International Statistical Classification of Diseases and Related Health Problems, 10th Revision codes: J41, J42, J43, and J44 for bronchitis, simple and chronic mucopurulent chronic bronchitis, unspecified chronic bronchitis, and emphysema respectively and I00-I99 for diseases of the cardiovascular system in patients greater than 25 years of age [20]. These codes have also been implemented in previous studies [17,21], and this methodology has been validated and employed in previous studies analyzing mortality data [21–23]. Our study was exempt from institutional review board approval, as we utilized a de-identified government-provided public-use dataset in accordance with Strengthening the Reporting of Observational Studies in Epidemiology (STROBE) guidelines [24].

### Data abstraction

Our demographic variables were based on population size, age distribution, sex composition, ethnoracial background, geographic location, urbanization level, and place of death from 1999 to 2020, whereas locations of death encompassed inpatient facilities, outpatient clinics, emergency rooms, cases of sudden death, residences, hospice/nursing homes, long-term care facilities, and instances where the location remained unspecified. Ethnoracial categories included Hispanic (Latino), Non-Hispanic (NH) White, NH Black/African American, NH American Indian/Alaskan Native, and NH Asian. These classifications are consistent with those used in prior analyses from the CDC WONDER database and follow the guidelines set by the US Office of Management and Budget for reporting data on death certificates [25].

Furthermore, we categorized patients into fifteen-year intervals, including 25–39 years, 40–54 years, 55–69 years, 70–64 years, and 85+ years of age as utilized in previous studies [21]. To stratify the population geographically, we employed the Urban-Rural Classification Scheme from the National Center for Health Statistics and divided the counties into urban (large metropolitan area; population greater than 1 million), medium/small metropolitan area (population between 50,000–999,999), and rural (population lesser than 50,000) categories [18]. Additionally, we divided the United States into four regions based on the US Census Bureau’s classification: Northeast, Midwest, South, and West [26].

### Statistical analysis

We analyzed sex, ethnoracial, age, urbanization, and census-related patterns by computing both crude and age-adjusted mortality rates (AAMR) per 100,000 individuals. AAMR was age-standardized to the 2000 US population using direct standardization, a widely recognized technique that facilitates the comparison of mortality rates by adjusting for age-related differences in the population [27]. Temporal trends in AAMR for both CVD and COPD-related mortality were evaluated by fitting log-linear regression models using Joinpoint Regression Program (Version 5.0.2, National Cancer Institute) [28]. Joinpoint regression was employed to detect inflection points in the AAMR trends from 1999 to 2020, following established methodological guidelines [29]. According to these guidelines, for datasets with 17 to 21 time points, a maximum of three inflection points is recommended. Given that this study spans 22 years, the Joinpoint regression software was configured to identify up to four inflection points where significant changes in trend occurred. However, fewer inflection points were identified when the variation between trends was more pronounced with fewer joinpoints. Thus, the analysis permitted the identification of between 0 and 4 joinpoints. The Grid Search method (2, 2, 0), coupled with a permutation test and a parametric method, was used to estimate the annual percent change (APC) and its corresponding 95% confidence intervals (CIs). APC reflects the rate of change in AAMR over time, showing whether mortality rates are increasing or decreasing annually. A positive APC suggests an increase in mortality rates, while a negative APC indicates a decrease. APC values were considered statistically significant if their 95% CIs did not include zero, based on two-tailed t-tests, with statistical significance set at P ≤  0.05.

## Results

Between 1999 and 2020, there were 3,590,124 deaths related to both CVD and COPD among individuals aged 25 years and older in the US (S1 Table). The place of death was recorded for 3,449,936 of these cases, with the following distribution: 41.7% in medical facilities, 29.4% in the decedents’ homes, 21.4% in nursing homes or long-term care facilities, and 3.6% in hospices (S2 Table).

### Demographic trends in mortality

**Fig 1:**
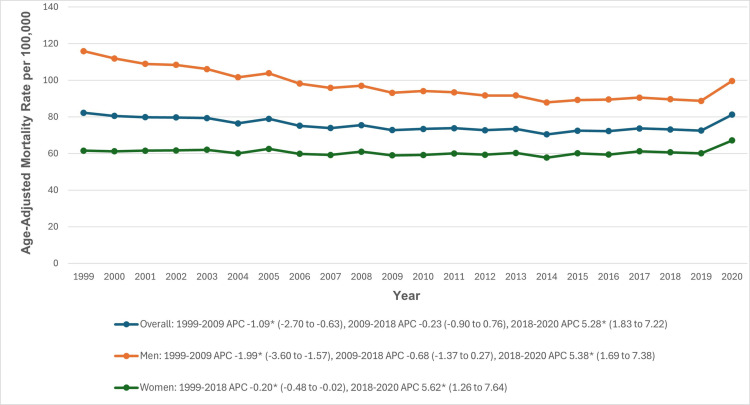
Overall and sex-stratified cardiovascular disease and chronic obstructive pulmonary disease-related age-adjusted mortality rates per 100,000 in adults in the United States, 1999 to 2020. *Indicates that the annual percentage change (APC) is significantly different from zero at α =  0.05. AAMR, age-adjusted mortality rate.

The AAMR was 82.2 in 1999 and slightly decreased to 81.2 in 2020. From 1999 to 2009, the AAMR experienced a significant decline from 82.2 to 72.8 (APC: −1.09; 95% CI: −2.70 to −0.63). This was followed by a period of stability from 2009 to 2018 (APC: −0.23; 95% CI: −0.90 to 0.76). However, from 2018 to 2020, the AAMR significantly increased to 81.2 (APC: 5.28; 95% CI: 1.83 to 7.22) (Fig 1, S3 and S4 Tables).

### Sex stratification

During the study period, men had higher AAMRs than women (96.5 vs 60.7). For men, the AAMR was 115.9 in 1999, which significantly declined until 2009 (APC: −1.99; 95% CI: −3.60 to −1.57). This was followed by a stable period from 2009 to 2018, and a steep increase from 2018 to 2020 (APC: 5.38; 95% CI: 1.69 to 7.38). Similarly, women’s AAMR was 61.5 in 1999, which significantly decreased until 2018 (APC: −0.20; 95% CI: −0.48 to −0.02), followed by a significant rise from 2018 to 2020 (APC: 5.62; 95% CI: 1.26 to 7.64) (Fig 1, S3 and S4 Tables).

### Stratification by age groups

**Fig 2:**
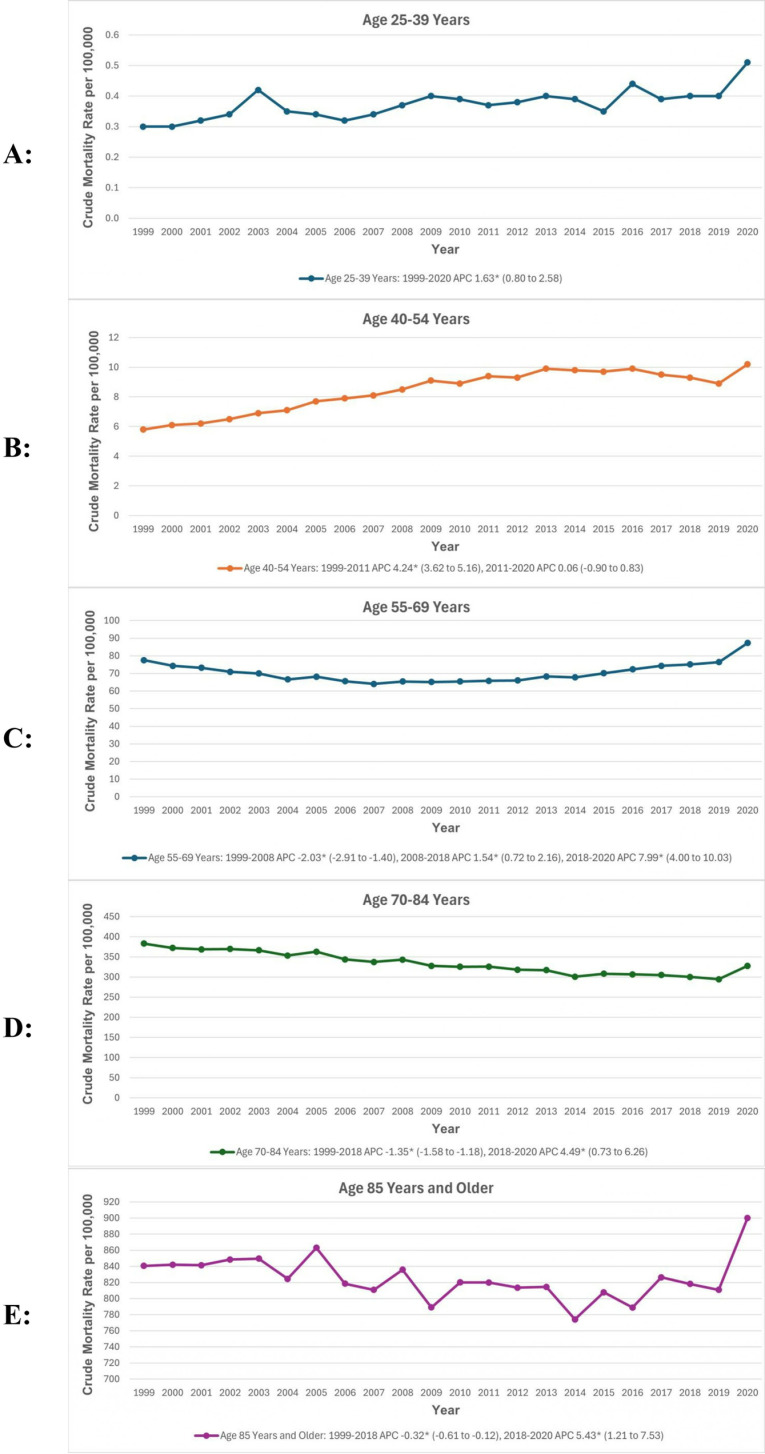
(A–E) Cardiovascular disease and chronic obstructive pulmonary disease-related age-adjusted mortality rates per 100,000, stratified by age groups in adults in the United States, 1999 to 2020. *Indicates that the annual percentage change (APC) is significantly different from zero at α =  0.05. AAMR, age-adjusted mortality rate; APC, annual percent change; CI, confidence interval.

When stratified by age groups, individuals aged 85 years and above had the highest crude mortality rates (824.1), while those aged 25–39 years had the lowest (0.4). For people aged 25–39 years, crude rates rose significantly from 1999 to 2020 (APC: 1.63; 95% CI: 0.80 to 2.58). For those aged 40–54 years, the rates significantly increased from 1999 to 2011 (APC: 4.24; 95% CI: 3.62 to 5.16). For individuals aged 55–69 years, the rates significantly declined from 1999 to 2008 (APC: −2.03; 95% CI: −2.91 to −1.40), followed by an increase until 2018 (APC: 1.54; 95% CI: 0.72 to 2.16), and a steep uptrend until 2020 (APC: 7.99; 95% CI: 4.00 to 10.03). For older adults aged 70–84 years and 85 years and above, rates decreased until 2018 (70–84 years APC: −1.35; 95% CI: −1.58 to −1.18; 85 years and above APC: −0.32; 95% CI: −0.61 to −0.11), followed by a steep increase from 2018 to 2020 (70–84 years APC: 4.49; 95% CI: 0.73 to 6.26; 85 years and above APC: 5.43; 95% CI: 1.21 to 7.53). (Fig 2, S3 and S5 Tables).

### Ethnoracial stratification

**Fig 3:**
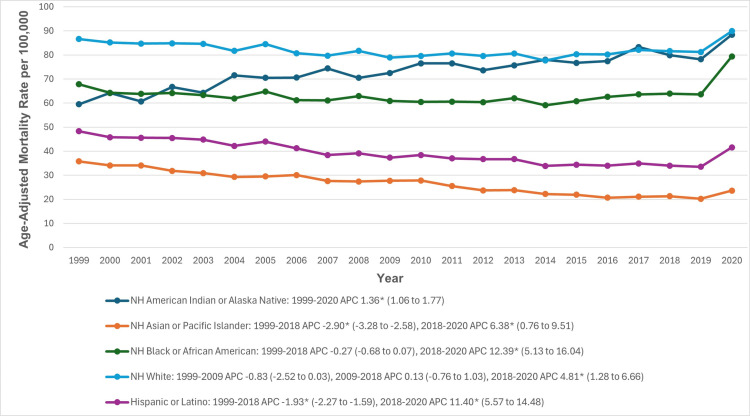
Cardiovascular disease and chronic obstructive pulmonary disease-related age-adjusted mortality rates per 100,000, stratified by ethnoracial groups in adults in the United States, 1999 to 2020. *Indicates that the annual percentage change (APC) is significantly different from zero at α =  0.05. AAMR, age-adjusted mortality rate; APC, annual percent change; CI, confidence interval.

When stratified by ethnoracial groups, the AAMRs were highest among NH white and lowest among NH Asian or Pacific Islander individuals (82.0 vs 25.1). The AAMRs for NH American Indian or Alaska Native individuals increased significantly from 1999 to 2020 (APC: 1.36; 95% CI: 1.06 to 1.77). Among NH white population, the AAMRs remained somewhat stable from 1999 to 2018, followed by a significant increase from 2018 to 2020 (APC: 4.81; 95% CI: 1.28 to 6.67). For NH Asian and Hispanic individuals, the AAMRs decreased significantly between 1999 and 2018 (NH Asian APC: −2.90; 95% CI: −3.28 to −2.58; Hispanic APC: −1.93; 95% CI: −2.27 to −1.59), followed by a significant increase from 2018 to 2020 (NH Asian APC: 6.38; 95% CI: 0.76 to 9.51; Hispanic APC: 11.40; 95% CI: 5.57 to 14.48). Similarly, among NH Black individuals, the rates remained stable until 2018, followed by a steep increase from 2018 to 2020 (APC: 12.39; 95% CI: 5.13 to 16.04) (Fig 3, S3 and S6 Tables).

### State-wise distribution

**Fig 4:**
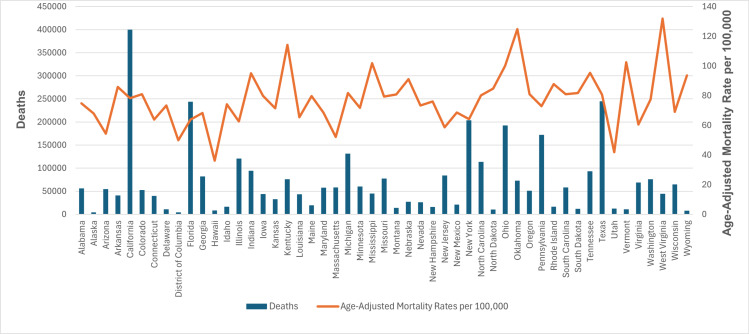
Cardiovascular disease and chronic obstructive pulmonary disease-related age-adjusted mortality rates per 100,000, stratified by state in adults in the United States, 1999 to 2020.

AAMR values varied significantly by state, ranging from 36.2 in Hawaii to 131.9 in West Virginia. States in the top 90th percentile (Ohio, Mississippi, Vermont, Kentucky, Oklahoma, West Virginia) had AAMRs about three to four times as high as those in the bottom 10th percentile (New Jersey, Arizona, Massachusetts, District of Columbia, Utah, Hawaii) (Fig 4 and S7 Table).

### Census region

**Fig 5:**
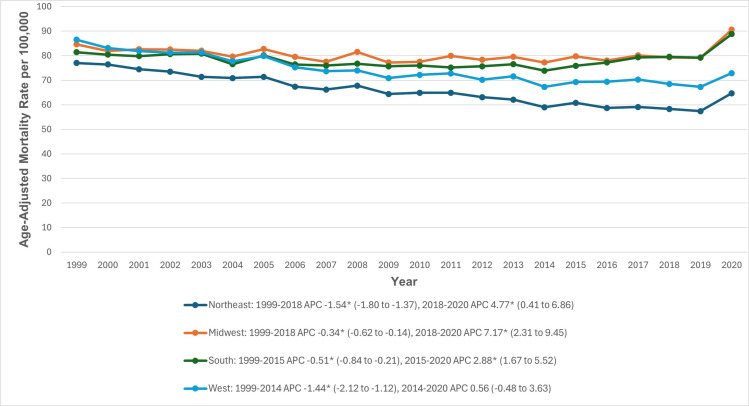
Cardiovascular disease and chronic obstructive pulmonary disease-related age-adjusted mortality rates per 100,000, stratified by census regions in adults in the United States, 1999 to 2020. *Indicates that the annual percentage change (APC) is significantly different from zero at α =  0.05. AAMR, age-adjusted mortality rate; APC, annual percent change; CI, confidence interval.

During the study period, the highest AAMRs were in the Midwestern region and the lowest were in the Northeastern region (80.5 vs 65.7). In both the Northeastern and Midwestern regions, the AAMRs decreased significantly between 1999 and 2018 (Northeast APC: −1.54; 95% CI: −1.80 to −1.37; Midwest APC: −0.34; 95% CI: −0.62 to −0.14), followed by a steep increase from 2018 to 2020 (Northeast APC: 4.77; 95% CI: 0.41 to 6.86; Midwest APC: 7.17; 95% CI: 2.31 to 9.45). Similarly, in the Southern region, the AAMRs decreased significantly between 1999 and 2015 (APC: −0.51; 95% CI: −0.84 to −0.21), followed by a significant increase between 2015 and 2020 (APC: 2.88; 95% CI: 1.67 to 5.52). In the Western region, the AAMRs also had a significant decrease between 1999 and 2014 (APC: −1.44; 95% CI: −2.12 to −1.12), followed by a period of stability until 2020. (Fig 5, S3 and S8 Tables).

### Urbanization

**Fig 6:**
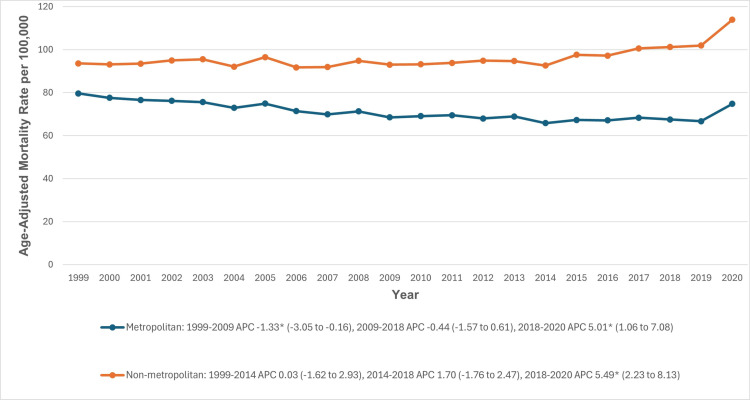
Cardiovascular disease and chronic obstructive pulmonary disease-related age-adjusted mortality rates per 100,000 in adults in the metropolitan and non-metropolitan areas in the United States, 1999 to 2020. *Indicates that the APC is significantly different from zero at α =  0.05. AAMR, age-adjusted mortality rate; APC, annual percent change; CI, confidence interval.

Throughout the study period, non-metropolitan areas had slightly higher AAMRs for CVD and COPD-related mortality compared to metropolitan areas (96.2 vs 70.9). In metropolitan areas, AAMRs initially decreased significantly from 1999 to 2009 (APC: −1.33; 95% CI: −3.05 to −0.16), followed by a stable period until 2018, and then a significant increase from 2018 to 2020 (APC: 5.01; 95% CI: 1.06 to 7.08). Similarly, in non-metropolitan areas, the AAMRs remained relatively stable until 2018, followed by a significant increase from 2018 to 2020 (APC: 5.49; 95% CI: 2.23 to 8.13) (Fig 6, S3 and S9 Tables).

### Mortality rates by individual disease type

#### Cardiovascular diseases.

**Fig 7:**
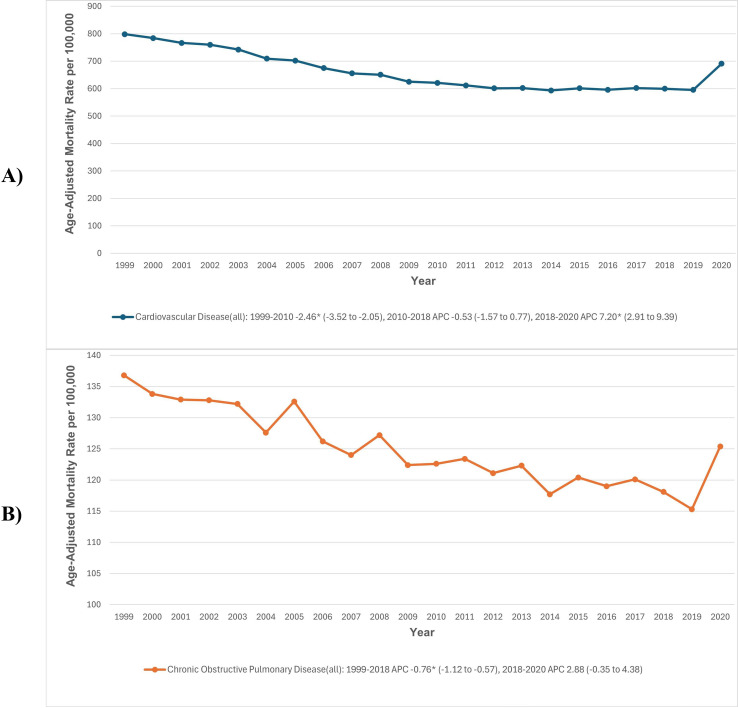
(A) Cardiovascular disease and (B) chronic obstructive pulmonary disease-related mortality in the United States, 1999–2020. *Indicates that the APC is significantly different from zero at α =  0.05. AAMR, age-adjusted mortality rate; APC, annual percent change; CI, confidence interval.

The AAMR due to CVDs was 798.5 in 1999 and decreased to 691.0 in 2020. The AAMRs due to CVD initially decreased significantly from 1999 to 2010 (APC: −2.46; 95% CI: −3.52 to −2.05), followed by a period of stability till 2018. This was followed by a steep increase in AAMR from 2018 to 2020 (APC: 7.20; 95% CI: 2.91 to 9.39) (Fig 7A, S3 and S10 Tables).

#### Chronic obstructive pulmonary disease alone.

The AAMR for COPD in 1999 was 136.8, which decreased slightly to 125.4 in 2020. The AAMRs for COPD initially decreased significantly from 1999 to 2018 (APC: −0.76; 95% CI: −1.12 to −0.57). This was followed by a non-significant increase from 2018 to 2020 (APC: 2.88; 95% CI: −0.35 to 4.38) (Fig 7B, S3 and S10 Tables).

### Age-adjusted mortality rates in 2021 and 2022

The AAMR for CVDs increased from 691 in 2020 to 728.1 in 2021, before decreasing to 674.6 in 2022. For COPD, the AAMR was 125.4 in 2020, 124.9 in 2021, and 119.3 in 2022, showing a slight decline over the years. The AAMR for combined CVD and COPD-related mortality varied slightly, with rates of 81.2 in 2020, 82 in 2021, and 78.8 in 2022.

## Discussion

Our analysis of mortality rates in the US population among adults (≥25 years) from 1999 to 2020 indicated a significant increase in mortality in recent years, despite an overall decline across various demographic groups, including sex, race/ethnicity, geographical region, and urbanization status. Older adults (≥85 years) had consistently higher AAMRs than their younger counterparts, and NH white individuals had the highest AAMRs among all the ethnoracial groups. Significant differences were also observed between metropolitan and non-metropolitan areas, with non-metropolitan areas having higher AAMRs for CVD and COPD-related mortality than metropolitan areas.

The incidence and prevalence of COPD and CVD have increased over the past two decades [30–32], reflecting broader trends in aging populations and the persistence of key risk factors such as smoking, hypertension, and obesity [33–36]. National health surveys and epidemiological studies have consistently reported rising rates of both diseases, particularly among older adults, with COPD being a leading cause of disability and CVD contributing to the majority of deaths in individuals over 65 [37]. Temporal trends in the prevalence of these conditions have varied by age, sex, and race/ethnicity, with a marked increase in diagnoses among older adults, as the number of individuals aged 65 and older is expected to nearly double by 2050 [32,38]. Furthermore, geographic disparities exist, with rural populations experiencing a higher burden due to limited access to healthcare and higher prevalence of smoking and other risk factors [39].

The present analysis revealed a complex trajectory of AAMRs with a pattern of initial decline followed by periods of increase. From 1999 to 2009, the AAMR experienced a significant decrease, which was followed by a period of stability from 2009 to 2018. However, from 2018 to 2020, the AAMR significantly increased. Overall, the AAMR was 82.9 in 1999 and slightly decreased to 81.4 in 2020. The initial decline from 1999 to 2009 is in part due to reductions in the prevalence of smoking, hypertension, and elevated cholesterol levels during this period [40–42]. National public health such as anti-smoking initiatives, public education on heart-healthy diets, and increased physical activity, as well as improved access to healthcare services, likely contributed to these trends [43–45]. In addition, advances in medical treatments, such as the use of statins, angiotensin-converting enzyme (ACE) inhibitors, and beta-blockers for CVD, and the introduction of long-acting bronchodilators and inhaled corticosteroids for COPD, have contributed to a reduction in disease-related mortality [46–49]. Public health and primary care interventions, including screening programs for hypertension and cholesterol management, smoking cessation programs, and increased access to chronic disease management, have further supported these improvements in health outcomes [50,51]. These efforts suggest that both primary and secondary prevention strategies for CVD and COPD have played an important role in decreasing mortality rates over the past two decades. Furthermore, the overall decline in CVD mortality during the late 20th century and early 2000s can be attributed to advancements in evidence-based medical and surgical treatments, as well as global prevention strategies aimed at reducing cardiovascular and pulmonary risk factors [52]. However, as the burden of chronic diseases like CVD and COPD shifted toward an aging population, the incidence of these conditions continued to rise, affecting older adults disproportionately.

However, our analysis indicates a concerning significant increase in recent AAMR trends from 2018 to 2020 with a much steeper increase in the year 2020. These trends were accompanied by a slight increase from 2020 to 2021, followed by a decline over the next year. Several factors contribute to this situation. First, the ongoing increase in the elderly population aged ≥ 75 years in economically advanced countries has led to a higher prevalence of chronic illnesses, increased morbidity, higher hospitalization rates, and more documented deaths due to CVD and COPD [53,54]. Second, our analysis indicates that this sharp rise coincides with the COVID-19 pandemic. During this time, medical resources were reallocated to prioritize COVID-19 patients, leading to decreased hospital admissions for cardiovascular conditions and limited access to cardiovascular and pulmonary care globally [54]. This resulted in inadequate treatment, delayed follow-ups, and a lack of timely attention to less urgent cases, causing patients to present in more deteriorated states and contributing to increased mortality. Third, patients with CVD and COPD often have comorbid conditions such as diabetes, hypertension, and heart failure. These comorbidities increase the risk of severe COVID-19, requiring intensive care, advanced hemodynamic monitoring, skilled nursing, and rehabilitation services, all of which were overwhelmed during the pandemic. This led to increased mortality, with approximately one in four individuals dying during hospitalization [55,56]. Fourth, growing evidence suggests that COVID-19 not only exacerbated pre-existing cardiovascular conditions but also contributed to an increased risk of subsequent cardiovascular events. Specifically, studies have shown that COVID-19 can act as a coronary artery disease risk equivalent, further raising the burden of CVD in affected populations [57]. Fifth, CVD and COPD patients, aware of their heightened vulnerability, might have avoided hospital visits out of fear of contracting COVID-19 [58,59]. Sixth, patients with chronic conditions like CVD and COPD faced significant challenges during the pandemic. The strain on healthcare systems and the scarcity of medical resources resulted in suboptimal health service provision. Additionally, difficulties in accessing essential medications and supplies, such as bronchodilators, oxygen therapy, antihypertensive drugs, and home health aides, led to inadequate management of these conditions [55]. Importantly, emerging research suggests that COVID-19 may also increase the risk of subsequent COPD morbidity and mortality, though this remains less explored. The exacerbation of pulmonary complications in COVID-19 patients and the interruption of regular care have likely contributed to worsened outcomes for individuals with COPD [60,61]. The long-term implications of these disruptions are significant, as they may contribute to enduring health challenges in the affected populations, underscoring the need for comprehensive post-pandemic healthcare strategies. Future research should focus on understanding the prolonged effects of delayed care and comorbidity exacerbation on mortality trends, particularly for vulnerable groups.

It is worth mentioning that the analysis of individual disease types revealed that AAMRs for CVD follow similar trends to those observed in combined disease data. However, COPD showed a non-significant increase from 2018 to 2020, followed by a consistent decline in subsequent years. The alignment of CVD trends with those of combined diseases may be attributed to the fact that most deaths in patients with both COPD and CVD are of cardiovascular origin.

Our analysis revealed that older adults consistently had the highest AAMRs, which is expected given the higher prevalence of CVD and COPD in this age group [62,63]. The higher prevalence of hypertension in elderly patients further contributes to the development of various CVDs [62]. The significant increase in mortality rates among middle-aged individuals since 2007 and older adults since 2018 underscores the necessity for targeted interventions that emphasize the early detection and management of risk factors such as hypertension, diabetes, and smoking. This rise may be due to deteriorating cardiovascular and pulmonary health and potential gaps in healthcare access and management for these groups [64,65]. Additionally, the global demographic landscape has seen a notable increase in the aging population. In 1990, individuals over 60 years old comprised 9.2% of the global population, which increased to 11.7% by 2013, with projections indicating a rise to 21.1% by 2050 [66]. This demographic shift is particularly relevant to CVD and COPD, which predominantly affect the elderly, suggesting an increasing impact on this age group [67]. As these conditions are prevalent and pose a substantial public health challenge worldwide, raising public awareness and enhancing management strategies for CVD and COPD, especially among vulnerable populations, has become an urgent global priority.

The finding of ethnoracial differences at the intersection of CVD and COPD was unexpected. Previous studies have shown that Black individuals with COPD tend to have shorter 6-minute walk distances, experience more severe exacerbations, have a lower health-related quality of life, and experience higher mortality rates [68,69]. Ethnoracial disparities regarding higher mortality rates among the Black population are also evident in studies involving CVD patients [70]. Based on this, we anticipated that Black individuals would experience the AAMRs related to these conditions among all ethnoracial groups. Surprisingly, our analysis indicated that the AAMRs were highest among NH White and lowest among NH Asian or Pacific Islander individuals. It is important to highlight that in 1990, there were pronounced mortality differences between Black and White Americans. For most age groups, Black Americans living in the highest-income areas of the US had significantly higher mortality rates than White Americans in the lowest-income areas. Since 1990, Black Americans have experienced substantial improvements in mortality across all age ranges and in both higher- and lower-income areas. Given that Black Americans are more likely to reside in lower-income counties, the gains in these counties have had a significant impact on reducing the racial life expectancy gap. These reductions in mortality were substantial enough to narrow the racial mortality gap by 48.9%, even though White Americans also experienced mortality improvements. Between 1990 and 2018, the life expectancy gap between Black and White Americans in the US decreased from 7.0 to 3.6 years [71]. Despite this progress, the higher mortality rates among NH White population observed in our study is an unusual finding that warrants further exploration. Future research should continue to explore these dynamics to inform more effective and equitable health interventions. Addressing these disparities requires a multifaceted strategy that goes beyond ethnoracial categorization to include the specific contextual factors influencing health outcomes in different populations.

Our analysis revealed substantial disparities among various regions of the United States. The highest AAMRs were in the Midwestern region and the lowest were in the Northeastern region. Furthermore, states in the top 90th percentile (Ohio, Mississippi, Vermont, Kentucky, Oklahoma, and West Virginia) had AAMRs about three to four times as high as those in the bottom 10th percentile. Such disparities are likely to be multifactorial due to a different regional distribution of cardiovascular risk factors, healthcare access, local medical resources, and the availability of advanced medical services [72]. Furthermore, throughout the study period, non-metropolitan areas exhibited slightly higher AAMRs for these conditions compared to metropolitan areas. The geographic isolation of non-metropolitan regions poses significant challenges for accessing medical care, which is further exacerbated by socioeconomic disparities. Residents in these areas must travel longer distances to reach healthcare facilities, and the availability of healthcare practitioners is markedly lower than in metropolitan regions. Cultural and behavioral factors, such as higher smoking rates, combined with the shortage of healthcare professionals, contribute to disparities in health outcomes and access to care. Despite efforts by governmental and non-profit organizations to mitigate these disparities, the gaps continue to widen [72,73].

The intersection of CVD and COPD, highlighting that COPD patients often have other associated conditions including CVD is an area of particular interest given the recent attention to the presence of a chronic systemic inflammatory syndrome that hypothesizes the role of a chronic inflammatory process resulting in the development of multiple chronic diseases [74]. The present analysis further contributes to this interesting area of knowledge concerning patients suffering from both CVD and COPD. Nevertheless, given the substantial mortality associated with these conditions, there is an urgent need for population-wide policy measures to address disparities and improve early management. Healthcare authorities should emphasize efforts to prevent, recognize, and treat these conditions effectively while promoting prevention strategies across communities. This involves reducing the prevalence of risk factors such as obesity, smoking, poor diet, and physical inactivity. Additionally, actively tracking and mitigating modifiable and non-genetic risk factors for CVD and COPD can enhance population health and promote equity across diverse communities.

### Limitations

Our analysis of death rates faces several constraints, presenting opportunities for further research. First, reliance on nationwide databases introduces potential miscoding, affecting accuracy. The impact of changes in death certificate quality on CVD and COPD deaths is unclear, as is whether AAMR changes are due to disease incidence or case-fatality rates. Second, ICD-10 codes in the CDC WONDER datasets mainly capture diagnostic information, potentially omitting crucial mortality factors like comorbidities and treatment protocols, limiting analysis depth. Third, the CDC WONDER dataset lacks data on prior cardiovascular and pulmonary history, heart and lung function, and competing causes of death. Death certificates may underestimate cardiovascular and pulmonary mortality in younger subjects, complicating findings. Despite these limitations, death certificates remain valuable for studying CVD and COPD trends. Fourth, the absence of clinical and laboratory data in the CDC WONDER database prevented detailed subgroup analyses based on factors like socioeconomic status, comorbid conditions, and medication adherence. This knowledge gap hinders understanding of changing death rates and treatment effectiveness. Fifth, a key limitation of our study is the lack of specific prevalence data for COPD and CVD, as the analysis focused solely on mortality trends due to the constraints of the available database. Additionally, the reliance on death certificate data may not fully capture the full spectrum especially of asymptomatic cardiovascular conditions. Finally, socioeconomic factors, critical to health outcomes, were not considered, affecting our understanding of healthcare access and overall well-being.

## Conclusions

The mortality rates among the adult population with coexisting CVD and COPD vary. AAMRs remained stable from 2009 to 2018, following a significant decline between 1999 and 2009. However, there was a subsequent surge beginning in 2018, which significantly accelerated towards the end of the study period, particularly in 2020. This sharp increase in mortality in 2020 coincides with the onset of the COVID-19 pandemic, which likely exacerbated existing risk factors and health disparities. Elevated rates were observed predominantly among older adults, NH white natives, males, individuals residing in the Midwest region of the United States, and those dwelling in non-urban areas. These findings underscore the pressing need for systemic reforms in healthcare practices and protocols to address both pre-existing conditions and the ongoing impact of the pandemic.

## Supporting information

S1 Table Cardiovascular disease and chronic obstructive pulmonary disease-related mortality, stratified by sex and race in adults in the United States, 1999 to 2020. NH, non-Hispanic.
(DOCX)

S2 Table Cardiovascular disease and chronic obstructive pulmonary disease-related mortality, stratified by place of death in adults in the United States, 1999 to 2020. (DOCX)

S3 Table Annual percent change (APC) of cardiovascular disease and chronic obstructive pulmonary disease–related age-adjusted mortality rates per 100,000 in adults in the United States, 1999 to 2020. APC, annual percent change; NH, non-Hispanic; CVD, cardiovascular disease; COPD, chronic obstructive pulmonary disease.
(DOCX)

S4 Table Overall and sex‐stratified cardiovascular disease and chronic obstructive pulmonary disease–related age-adjusted mortality rates per 100,000 in adults in the United States from 1999 to 2020. (DOCX)

S5 Table Cardiovascular disease and chronic obstructive pulmonary disease-related mortality, stratified by age group in adults in the United States, 1999 to 2020. (DOCX)

S6 Table Cardiovascular disease and chronic obstructive pulmonary disease related age-adjusted mortality rates per 100,000 stratified by race in adults in the United States from 1999 to 2020. NH, non-Hispanic.
(DOCX)

S7 Table Cardiovascular disease and chronic obstructive pulmonary disease related age-adjusted mortality rates per 100,000, stratified by state in adults in the United States, 1999 to 2020. (DOCX)

S8 Table Cardiovascular disease and chronic obstructive pulmonary disease related age-adjusted mortality rate per 100,000 stratified by census region in adults in the United States 1999-2020. (DOCX)

S9 Table Overall cardiovascular disease and chronic obstructive pulmonary disease–related age-adjusted mortality rates per 100,000 in adults in the metropolitan and non-metropolitan areas in the United States, 1999 to 2020. (DOCX)

S10 Table Overall cardiovascular disease (CVD) alone and chronic obstructive pulmonary disease (COPD) alone–related age-adjusted mortality rates per 100,000 in the United States, 1999 to 2020. (DOCX)

## Abbreviations

APC: annual percentage change
AAMR: age-adjusted mortality rates
COPD: chronic obstructive pulmonary disease
CVD: cardiovascular disease
NH: non-Hispanic
