# Supplementary material for: Mortality trends and disparities for coexisting chronic obstructive pulmonary disease and cardiovascular disease: A retrospective analysis of deaths in the United States from 1999–2020
Source: PLoS One. 2025 Feb 4;20(2):e0317592. doi: 10.1371/journal.pone.0317592 (PMC11793733; doi:10.1371/journal.pone.0317592)
Supplement: S1 Table — NH, non-Hispanic. (DOCX) [file pone.0317592.s001.docx]

**S1 Table.** Cardiovascular Disease and Chronic Obstructive Pulmonary Disease-related Mortality, Stratified by Sex and Race in Adults in the United States, 1999 to 2020.

| Deaths | | | | | | | | | |
| --- | --- | --- | --- | --- | --- | --- | --- | --- | --- |
| Year | **Overall** | **Women** | **Men** | **NH White** | **NH Black or African American** | **NH Asian or Pacific Islander** | **NH American Indian or Alaska Native** | **Hispanic or Latino** | **Population** |
| 1999 | 145185 | 66087 | 79098 | 129680 | 9773 | 1265 | 400 | 3633 | 180408769 |
| 2000 | 143709 | 66480 | 77229 | 128555 | 9401 | 1255 | 469 | 3615 | 181984640 |
| 2001 | 144685 | 67680 | 77005 | 129103 | 9507 | 1372 | 456 | 3856 | 184305128 |
| 2002 | 146668 | 68590 | 78078 | 130650 | 9739 | 1363 | 497 | 4006 | 186208028 |
| 2003 | 148142 | 69899 | 78243 | 131892 | 9780 | 1408 | 518 | 4160 | 188090429 |
| 2004 | 144771 | 68399 | 76372 | 128594 | 9764 | 1403 | 589 | 4136 | 190205384 |
| 2005 | 152313 | 72320 | 79993 | 134800 | 10513 | 1520 | 606 | 4608 | 192551384 |
| 2006 | 147835 | 70216 | 77619 | 130552 | 10212 | 1647 | 617 | 4524 | 195019359 |
| 2007 | 148267 | 70630 | 77637 | 130875 | 10432 | 1593 | 667 | 4486 | 197403777 |
| 2008 | 154598 | 73974 | 80624 | 136113 | 11034 | 1675 | 687 | 4808 | 199795090 |
| 2009 | 152141 | 72602 | 79539 | 133383 | 11110 | 1781 | 734 | 4849 | 202107016 |
| 2010 | 155861 | 74000 | 81861 | 136409 | 11282 | 1875 | 773 | 5205 | 203891983 |
| 2011 | 161151 | 76793 | 84358 | 140960 | 11726 | 1885 | 839 | 5419 | 206592936 |
| 2012 | 162965 | 77547 | 85418 | 141944 | 12173 | 1893 | 841 | 5699 | 208826037 |
| 2013 | 168942 | 80492 | 88450 | 146531 | 12980 | 2043 | 917 | 6054 | 211085314 |
| 2014 | 166252 | 78647 | 87605 | 143741 | 12890 | 2060 | 1011 | 5991 | 213809280 |
| 2015 | 175366 | 83738 | 91628 | 151207 | 13764 | 2190 | 1052 | 6479 | 216553817 |
| 2016 | 178805 | 84334 | 94471 | 153494 | 14665 | 2189 | 1130 | 6762 | 218641417 |
| 2017 | 187373 | 89049 | 98324 | 160303 | 15436 | 2386 | 1254 | 7314 | 221447331 |
| 2018 | 190566 | 90177 | 100389 | 162623 | 16044 | 2541 | 1260 | 7478 | 223311190 |
| 2019 | 193401 | 91263 | 102138 | 164795 | 16522 | 2535 | 1303 | 7702 | 224981167 |
| 2020 | 221128 | 103705 | 117423 | 184663 | 21170 | 3143 | 1542 | 10007 | 226635013 |
| Total | 3590124 | 1696622 | 1893502 | 3130867 | 269917 | 41022 | 18162 | 120791 | 4473854489 |

NH, non-Hispanic.
