# Supplementary material for: Mortality trends and disparities for coexisting chronic obstructive pulmonary disease and cardiovascular disease: A retrospective analysis of deaths in the United States from 1999–2020
Source: PLoS One. 2025 Feb 4;20(2):e0317592. doi: 10.1371/journal.pone.0317592 (PMC11793733; doi:10.1371/journal.pone.0317592)
Supplement: S2 Table — (DOCX) [file pone.0317592.s002.docx]

**S2 Table.** Cardiovascular Disease and Chronic Obstructive Pulmonary Disease-related Mortality, Stratified by Place of Death in Adults in the United States, 1999 to 2020

| **Deaths** | | | | |
| --- | --- | --- | --- | --- |
| **Year** | **Medical**  **Facility** | **Nursing Home/Long-term**  **Care Facility** | **Hospices** | **Home** |
| 1999 | 79581 | 32543 | N/A | 29732 |
| 2000 | 76699 | 32929 | N/A | 30591 |
| 2001 | 76210 | 33134 | N/A | 31236 |
| 2002 | 75609 | 33642 | N/A | 32797 |
| 2003 | 73851 | 33952 | 179 | 34786 |
| 2004 | 70239 | 33337 | 355 | 35031 |
| 2005 | 72523 | 35089 | 1460 | 37673 |
| 2006 | 68482 | 33772 | 2104 | 38133 |
| 2007 | 67378 | 33226 | 3209 | 39126 |
| 2008 | 68463 | 33903 | 4059 | 40703 |
| 2009 | 64097 | 32686 | 4467 | 41553 |
| 2010 | 64761 | 34073 | 5620 | 45066 |
| 2011 | 65619 | 34929 | 6525 | 47549 |
| 2012 | 63318 | 34646 | 7867 | 50211 |
| 2013 | 63887 | 35085 | 8349 | 53934 |
| 2014 | 61190 | 34501 | 9048 | 54960 |
| 2015 | 63330 | 36052 | 10970 | 58558 |
| 2016 | 61942 | 35957 | 11940 | 62007 |
| 2017 | 63727 | 38451 | 12634 | 65168 |
| 2018 | 62783 | 38332 | 13008 | 68699 |
| 2019 | 62550 | 37906 | 13632 | 71125 |
| 2020 | 71253 | 39472 | 13450 | 87313 |
| **Total** | 1497492 | 767617 | 128876 | 1055951 |
