# Supplementary material for: Mortality trends and disparities for coexisting chronic obstructive pulmonary disease and cardiovascular disease: A retrospective analysis of deaths in the United States from 1999–2020
Source: PLoS One. 2025 Feb 4;20(2):e0317592. doi: 10.1371/journal.pone.0317592 (PMC11793733; doi:10.1371/journal.pone.0317592)
Supplement: S3 Table — APC, annual percent change; NH, non-Hispanic; CVD, cardiovascular disease; COPD, chronic obstructive pulmonary disease. (DOCX) [file pone.0317592.s003.docx]

**S3 Table.** Annual percent change (APC) of Cardiovascular Disease and Chronic Obstructive Pulmonary Disease–related Age-Adjusted Mortality Rates per 100,000 in Adults in the United States, 1999 to 2020

| Year Interval | APC (95% CI) |
| --- | --- |
| Overall | |
| 1999-2009 | -1.09 (-2.70 to -0.63) |
| 2009-2018 | -0.23 (-0.90 to 0.76) |
| 2018- 2020 | 5.28 (1.83 to 7.22) |
| Men | |
| 1999-2009 | -1.99 (-3.60 to -1.57) |
| 2009-2018 | -0.68 (-1.37 to 0.27) |
| 2018-2020 | 5.38 (1.69 to 7.38) |
| Women | |
| 1999-2018 | -0.20 (-0.48 to -0.02) |
| 2018-2020 | 5.62 (1.26 to 7.64) |
| 25-39 years | |
| 1999-2020 | 1.63 (0.80 to 2.58) |
| 40-54 years | |
| 1999-2011 | 4.24 (3.62 to 5.16) |
| 2011-2020 | 0.06 (-0.90 to 0.83) |
| 55-69 years | |
| 1999-2008 | -2.03 (-2.91 to -1.40) |
| 2008-2018 | 1.54 (0.72 to 2.16) |
| 2018-2020 | 7.99 (4.00 to 10.03) |
| 70-84 years | |
| 1999-2018 | -1.35 (-1.58 to -1.18) |
| 2018-2020 | 4.49 (0.73 to 6.26) |
| 85 years and older | |
| 1999-2018 | -0.32 (-0.61 to -0.12) |
| 2018-2020 | 5.43 (1.21 to 7.53) |
| NH White | |
| 1999-2009 | -0.83 (-2.52 to 0.03) |
| 2009-2018 | 0.13 (-0.76 to 1.03) |
| 2018-2020 | 4.81 (1.28 to 6.66) |
| NH Black or African American | |
| 1999-2018 | -0.27 (-0.68 to 0.07) |
| 2018-2020 | 12.39 (5.13 to 16.04) |
| Hispanic or Latino | |
| 1999-2018 | -1.93 (-2.27 to -1.59) |
| 2018-2020 | 11.40 (5.57 to 14.48) |
| NH Asian or Pacific Islander | |
| 1999-2018 | -2.90 (-3.28 to -2.58) |
| 2018-2020 | 6.38 (0.76 to 9.51) |
| NH American Indian or Alaska Native | |
| 1999-2020 | 1.36 (1.06 to 1.77) |
| Non-metropolitan areas | |
| 1999-2014 | -0.03 (-1.62 to 2.93) |
| 2014-2018 | 1.70 (-1.76 to 2.47) |
| 2018-2020 | 5.49 (2.23 to 8.13) |
| Metropolitan area | |
| 1999-2009 | -1.33 (-3.05 to -0.16) |
| 2009-2018 | -0.44 (-1.57 to 0.61) |
| 2018-2020 | 5.01 (1.06 to 7.08) |
| Northeast | |
| 1999-2018 | -1.54 (-1.80 to -1.37) |
| 2018-2020 | 4.77 (0.41 to 6.86) |
| Midwest | |
| 1999-2018 | -0.34 (-0.62 to -0.14) |
| 2018-2020 | 7.17 (2.31 to 9.45) |
| South | |
| 1999-2015 | -0.51 (-0.84 to -0.21) |
| 2015-2020 | 2.89 (1.67 to 5.52) |
| West | |
| 1999-2014 | -1.44 (-2.12 to -1.12) |
| 2014-2020 | 0.56 (-0.48 to 3.63) |
| CVD Alone | |
| 1999-2010 | -2.46 (-3.52 to -2.05) |
| 2010-2018 | -0.53 (-1.57 to 0.77) |
| 2018-2020 | 7.20 (2.91 to 9.39) |
| COPD Alone | |
| 1999-2018 | -0.76 (-1.12 to -0.57) |
| 2018-2020 | 2.88 (-0.35 to 4.38) |

APC = Annual percent change; NH = non-Hispanic; CVD = Cardiovascular Disease; COPD = Chronic obstructive pulmonary disease
