# Supplementary material for: Mortality trends and disparities for coexisting chronic obstructive pulmonary disease and cardiovascular disease: A retrospective analysis of deaths in the United States from 1999–2020
Source: PLoS One. 2025 Feb 4;20(2):e0317592. doi: 10.1371/journal.pone.0317592 (PMC11793733; doi:10.1371/journal.pone.0317592)
Supplement: S4 Table — (DOCX) [file pone.0317592.s004.docx]

**S4 Table.** Overall and Sex‐Stratified Cardiovascular Disease and Chronic Obstructive Pulmonary Disease–related Age-Adjusted Mortality Rates per 100,000 in Adults in the United States from 1999 to 2020

| Age-Adjusted Rate (95% CI) | | | |
| --- | --- | --- | --- |
| Year | **Men** | **Women** | **Overall** |
| 1999 | 115.9 (115.1-116.7) | 61.5 (61.0-61.9) | 82.2 (81.8-82.6) |
| 2000 | 111.9 (111.1-112.7) | 61.2 (60.8-61.7) | 80.5 (80.1-80.9) |
| 2001 | 108.9 (108.1-109.7) | 61.5 (61.1-62.0) | 79.8 (79.4-80.2) |
| 2002 | 108.4 (107.7-109.2) | 61.7 (61.2-62.1) | 79.7 (79.3-80.2) |
| 2003 | 106.1 (105.4-106.9) | 62.0 (61.6-62.5) | 79.3 (78.9-79.7) |
| 2004 | 101.6 (100.9-102.4) | 60.1 (59.6-60.5) | 76.4 (76.0-76.8) |
| 2005 | 103.8 (103.0-104.5) | 62.5 (62.1-63.0) | 78.9 (78.5-79.3) |
| 2006 | 98.2 (97.5-98.9) | 59.8 (59.4-60.3) | 75.1 (74.8-75.5) |
| 2007 | 95.8 (95.1-96.5) | 59.2 (58.8-59.7) | 73.9 (73.6-74.3) |
| 2008 | 97.0 (96.4-97.7) | 61.0 (60.6-61.4) | 75.5 (75.2-75.9) |
| 2009 | 93.1 (92.4-93.7) | 59.0 (58.6-59.5) | 72.8 (72.5-73.2) |
| 2010 | 94.1 (93.4-94.7) | 59.2 (58.8-59.6) | 73.4 (73.0-73.8) |
| 2011 | 93.4 (92.8-94.0) | 60.0 (59.6-60.5) | 73.8 (73.4-74.2) |
| 2012 | 91.7 (91.1-92.3) | 59.3 (58.8-59.7) | 72.7 (72.3-73.0) |
| 2013 | 91.7 (91.1-92.3) | 60.3 (59.9-60.7) | 73.4 (73.1-73.8) |
| 2014 | 87.9 (87.3-88.5) | 57.7 (57.3-58.1) | 70.4 (70.0-70.7) |
| 2015 | 89.2 (88.7-89.8) | 60.1 (59.7-60.5) | 72.4 (72.1-72.8) |
| 2016 | 89.5 (89.0-90.1) | 59.4 (59.0-59.8) | 72.2 (71.9-72.5) |
| 2017 | 90.5 (89.9-91.1) | 61.2 (60.8-61.6) | 73.7 (73.4-74.0) |
| 2018 | 89.6 (89.0-90.2) | 60.6 (60.2-61.0) | 73.1 (72.8-73.4) |
| 2019 | 88.7 (88.2-89.3) | 60.1 (59.7-60.5) | 72.5 (72.1-72.8) |
| 2020 | 99.6 (99.0-100.1) | 67.1 (66.7-67.6) | 81.2 (80.9-81.6) |
| Overall | 96.5 (96.4-96.6) | 60.7 (60.6-60.7) | 75.4 (75.3-75.4) |
