# Supplementary material for: Mortality trends and disparities for coexisting chronic obstructive pulmonary disease and cardiovascular disease: A retrospective analysis of deaths in the United States from 1999–2020
Source: PLoS One. 2025 Feb 4;20(2):e0317592. doi: 10.1371/journal.pone.0317592 (PMC11793733; doi:10.1371/journal.pone.0317592)
Supplement: S5 Table — (DOCX) [file pone.0317592.s005.docx]

**S5 Table.** Cardiovascular Disease and Chronic Obstructive Pulmonary Disease-related Mortality, Stratified by Age group in Adults in the United States, 1999 to 2020.

|  | **Deaths** | | | | | **Crude Mortality Rate (95% CI)** | | | | |
| --- | --- | --- | --- | --- | --- | --- | --- | --- | --- | --- |
| **Year** | **25-39 Years** | **40-54 years** | **55-69 Years** | **70- 84 years** | **85 years and older** | **25-39 Years** | **40-54 years** | **55-69 Years** | **70- 84 years** | **85 years and older** |
| 1999 | 188 | 3431 | 25833 | 80814 | 34919 | 0.3 (0.3-0.3) | 5.8 (5.6-6) | 77.5 (76.6-78.5) | 383 (380.3-385.6) | 840.6 (831.8-849.4) |
| 2000 | 186 | 3695 | 25133 | 78999 | 35696 | 0.3 (0.3-0.3) | 6.1 (5.9-6.3) | 74.3 (73.4-75.3) | 372.3 (369.7-374.9) | 842 (833.2-850.7) |
| 2001 | 199 | 3878 | 25364 | 78956 | 36288 | 0.3 (0.3-0.4) | 6.2 (6-6.4) | 73.2 (72.3-74.1) | 368.7 (366.1-371.3) | 841.5 (832.8-850.1) |
| 2002 | 210 | 4102 | 25770 | 79512 | 37074 | 0.3 (0.3-0.4) | 6.5 (6.3-6.7) | 70.9 (70-71.8) | 369.6 (367-372.1) | 848.6 (840-857.2) |
| 2003 | 257 | 4426 | 26494 | 79020 | 37945 | 0.4 (0.4-0.5) | 6.9 (6.7-7.2) | 70 (69.2-70.9) | 366.4 (363.8-369) | 849.6 (841.1-858.2) |
| 2004 | 209 | 4577 | 26225 | 76279 | 37481 | 0.4 (0.3-0.4) | 7.1 (6.9-7.3) | 66.6 (65.8-67.4) | 353.4 (350.9-355.9) | 824.5 (816.2-832.9) |
| 2005 | 204 | 5051 | 27854 | 78694 | 40510 | 0.3 (0.3-0.4) | 7.7 (7.5-8) | 68.1 (67.3-68.9) | 363 (360.5-365.6) | 863.1 (854.7-871.6) |
| 2006 | 196 | 5202 | 27866 | 74741 | 39830 | 0.3 (0.3-0.4) | 7.9 (7.7-8.1) | 65.6 (64.8-66.4) | 343.7 (341.3-346.2) | 818.5 (810.5-826.6) |
| 2007 | 209 | 5359 | 28216 | 73622 | 40861 | 0.3 (0.3-0.4) | 8.1 (7.9-8.4) | 64 (63.2-64.7) | 337.5 (335-339.9) | 810.8 (802.9-818.7) |
| 2008 | 225 | 5600 | 29918 | 75429 | 43426 | 0.4 (0.3-0.4) | 8.5 (8.3-8.7) | 65.4 (64.7-66.1) | 343.1 (340.6-345.5) | 835.8 (827.9-843.6) |
| 2009 | 242 | 5987 | 30910 | 72646 | 42356 | 0.4 (0.4-0.5) | 9.1 (8.9-9.3) | 65.1 (64.4-65.8) | 327.8 (325.4-330.2) | 789.1 (781.6-796.7) |
| 2010 | 236 | 5858 | 31976 | 72737 | 45054 | 0.4 (0.3-0.4) | 8.9 (8.7-9.1) | 65.4 (64.7-66.1) | 325.6 (323.2-328) | 820.1 (812.6-827.7) |
| 2011 | 225 | 6159 | 33507 | 74219 | 47041 | 0.4 (0.3-0.4) | 9.4 (9.1-9.6) | 65.8 (65.1-66.5) | 325.8 (323.4-328.1) | 819.9 (812.5-827.3) |
| 2012 | 237 | 6087 | 34705 | 74040 | 47896 | 0.4 (0.3-0.4) | 9.3 (9.1-9.6) | 66 (65.3-66.7) | 318 (315.7-320.3) | 813.5 (806.3-820.8) |
| 2013 | 248 | 6386 | 36838 | 76266 | 49204 | 0.4 (0.4-0.5) | 9.9 (9.6-10.1) | 68.3 (67.6-69) | 317.1 (314.8-319.3) | 814.5 (807.3-821.7) |
| 2014 | 246 | 6264 | 37564 | 74474 | 47704 | 0.4 (0.3-0.4) | 9.8 (9.5-10) | 67.8 (67.1-68.5) | 300.8 (298.7-303) | 774.1 (767.2-781.1) |
| 2015 | 225 | 6150 | 39933 | 78275 | 50783 | 0.4 (0.3-0.4) | 9.7 (9.5-9.9) | 70.1 (69.4-70.8) | 308.1 (305.9-310.3) | 807.7 (800.7-814.8) |
| 2016 | 291 | 6189 | 42148 | 79849 | 50328 | 0.4 (0.4-0.5) | 9.9 (9.7-10.2) | 72.3 (71.6-73) | 306.6 (304.5-308.7) | 788.8 (781.9-795.7) |
| 2017 | 258 | 5922 | 43740 | 84001 | 53452 | 0.4 (0.3-0.4) | 9.5 (9.3-9.8) | 74.3 (73.7-75) | 304.9 (302.8-306.9) | 826.3 (819.3-833.3) |
| 2018 | 269 | 5717 | 44583 | 86448 | 53549 | 0.4 (0.4-0.5) | 9.3 (9.1-9.6) | 75.1 (74.4-75.8) | 300.2 (298.2-302.2) | 818.2 (811.3-825.2) |
| 2019 | 272 | 5414 | 45793 | 88370 | 53552 | 0.4 (0.4-0.5) | 8.9 (8.7-9.1) | 76.4 (75.7-77.1) | 294.6 (292.6-296.5) | 810.8 (803.9-817.7) |
| 2020 | 345 | 6183 | 52623 | 102036 | 59941 | 0.5 (0.5-0.6) | 10.2 (9.9-10.4) | 87.3 (86.6-88) | 327.8 (325.8-329.8) | 900.2 (893-907.4) |
| **Overall** | 5177 | 117637 | 742993 | 1739427 | 984890 | 0.4 (0.4-0.4) | 8.4 (8.4-8.5) | 70.7 (70.5-70.8) | 332 (331.5-332.5) | 824.1 (822.5-825.7) |
