# Supplementary material for: Mortality trends and disparities for coexisting chronic obstructive pulmonary disease and cardiovascular disease: A retrospective analysis of deaths in the United States from 1999–2020
Source: PLoS One. 2025 Feb 4;20(2):e0317592. doi: 10.1371/journal.pone.0317592 (PMC11793733; doi:10.1371/journal.pone.0317592)
Supplement: S6 Table — NH, non-Hispanic. (DOCX) [file pone.0317592.s006.docx]

**S6 Table.** Cardiovascular Disease and Chronic Obstructive Pulmonary Disease related Age-Adjusted Mortality Rates per 100,000 stratified by Race in Adults in the United States from 1999 to 2020

| Age-Adjusted Rate (95% CI) | | | | | |
| --- | --- | --- | --- | --- | --- |
| Year | **NH White** | **NH Black or African American** | **NH American Indian or Alaska Native** | **Hispanic or Latino** | **NH Asian or Pacific Islander** |
| 1999 | 86.6 (86.1-87.1) | 67.8 (66.4-69.1) | 59.5 (53.4-65.5) | 48.3 (46.6-49.9) | 35.8 (33.8-37.9) |
| 2000 | 85.2 (84.8-85.7) | 64.3 (62.9-65.6) | 64.2 (58.2-70.3) | 45.8 (44.3-47.4) | 34.1 (32.2-36.1) |
| 2001 | 84.7 (84.2-85.2) | 63.8 (62.5-65.1) | 60.7 (54.9-66.5) | 45.6 (44.1-47.1) | 34.1 (32.3-36) |
| 2002 | 84.8 (84.3-85.3) | 64.2 (62.9-65.5) | 66.7 (60.6-72.8) | 45.5 (44.0-46.9) | 31.8 (30.1-33.6) |
| 2003 | 84.6 (84.2-85.1) | 63.3 (62-64.6) | 64.3 (58.4-70.1) | 44.8 (43.4-46.2) | 30.9 (29.3-32.6) |
| 2004 | 81.7 (81.3-82.2) | 61.9 (60.6-63.1) | 71.5 (65.4-77.6) | 42.2 (40.9-43.5) | 29.3 (27.7-30.9) |
| 2005 | 84.5 (84.0-84.9) | 64.8 (63.6-66.1) | 70.5 (64.5-76.4) | 44.0 (42.7-45.3) | 29.5 (28-31) |
| 2006 | 80.7 (80.3-81.1) | 61.2 (60-62.4) | 70.6 (64.7-76.5) | 41.2 (39.9-42.4) | 30.1 (28.6-31.6) |
| 2007 | 79.7 (79.3-80.2) | 61.1 (59.9-62.3) | 74.4 (68.4-80.3) | 38.4 (37.2-39.5) | 27.6 (26.2-28.9) |
| 2008 | 81.7 (81.2-82.1) | 62.9 (61.7-64.1) | 70.5 (64.9-76.1) | 39.1 (38.0-40.3) | 27.4 (26.1-28.7) |
| 2009 | 78.9 (78.5-79.3) | 60.9 (59.7-62.1) | 72.5 (67-78.1) | 37.3 (36.2-38.4) | 27.7 (26.4-29) |
| 2010 | 79.6 (79.2-80.1) | 60.5 (59.4-61.6) | 76.5 (70.8-82.2) | 38.4 (37.3-39.4) | 27.8 (26.5-29) |
| 2011 | 80.6 (80.2-81.1) | 60.6 (59.5-61.8) | 76.5 (71-82) | 37.0 (36.0-38.0) | 25.5 (24.4-26.7) |
| 2012 | 79.6 (79.2-80.0) | 60.4 (59.3-61.5) | 73.6 (68.4-78.9) | 36.7 (35.7-37.7) | 23.7 (22.6-24.8) |
| 2013 | 80.6 (80.2-81.0) | 62.0 (60.9-63.1) | 75.7 (70.5-80.8) | 36.7 (35.8-37.7) | 23.8 (22.8-24.9) |
| 2014 | 77.7 (77.3-78.1) | 59.1 (58-60.1) | 78 (73-83.1) | 33.9 (33.0-34.8) | 22.2 (21.2-23.2) |
| 2015 | 80.3 (79.9-80.7) | 60.8 (59.8-61.8) | 76.7 (71.8-81.5) | 34.4 (33.6-35.3) | 21.9 (21-22.8) |
| 2016 | 80.2 (79.8-80.6) | 62.6 (61.5-63.6) | 77.4 (72.7-82.1) | 34.0 (33.2-34.9) | 20.7 (19.8-21.6) |
| 2017 | 82.1 (81.7-82.5) | 63.6 (62.6-64.7) | 83.3 (78.5-88.1) | 34.9 (34.1-35.7) | 21.1 (20.2-22) |
| 2018 | 81.6 (81.2-82.0) | 63.9 (62.9-65.0) | 79.9 (75.3-84.4) | 34.0 (33.2-34.8) | 21.3 (20.4-22.1) |
| 2019 | 81.2 (80.8-81.6) | 63.6 (62.6-64.6) | 78.2 (73.8-82.6) | 33.5 (32.8-34.3) | 20.2 (19.4-21) |
| 2020 | 89.9 (89.4-90.3) | 79.4 (78.3-80.5) | 88.4 (83.8-92.9) | 41.6 (40.8-42.5) | 23.6 (22.8-24.5) |
| Overall | 82.0 (81.9-82.1) | 63.6 (63.3-63.8) | 74.5 (73.4-75.7) | 38.1 (37.9-38.3) | 25.1 (24.8-25.3) |

NH=non-Hispanic
