# Supplementary material for: Mortality trends and disparities for coexisting chronic obstructive pulmonary disease and cardiovascular disease: A retrospective analysis of deaths in the United States from 1999–2020
Source: PLoS One. 2025 Feb 4;20(2):e0317592. doi: 10.1371/journal.pone.0317592 (PMC11793733; doi:10.1371/journal.pone.0317592)
Supplement: S7 Table — (DOCX) [file pone.0317592.s007.docx]

**S7 Table.** Cardiovascular Disease and Chronic Obstructive Pulmonary Disease related Age-Adjusted Mortality Rates per 100,000, Stratified by State in Adults in the United States, 1999 to 2020.

| State | Age-Adjusted Rate (95% CI) |
| --- | --- |
| Alabama | 74.8 (74.2-75.4) |
| Alaska | 67.9 (65.7-70.0) |
| Arizona | 54.4 (53.9-54.8) |
| Arkansas | 85.8 (84.9-86.6) |
| California | 78.2 (78.0-78.5) |
| Colorado | 80.9 (80.2-81.6) |
| Connecticut | 63.9 (63.3-64.6) |
| Delaware | 73.4 (72.0-74.8) |
| District of Columbia | 49.9 (48.3-51.4) |
| Florida | 63.9 (63.7-64.2) |
| Georgia | 68.3 (67.9-68.8) |
| Hawaii | 36.2 (35.4-37.0) |
| Idaho | 74.2 (73.0-75.3) |
| Illinois | 62.5 (62.2-62.9) |
| Indiana | 95.0 (94.4-95.6) |
| Iowa | 79.8 (79.0-80.5) |
| Kansas | 71.5 (70.7-72.3) |
| Kentucky | 114.2 (113.3-115.0) |
| Louisiana | 65.5 (64.9-66.1) |
| Maine | 79.6 (78.5-80.7) |
| Maryland | 68.5 (67.9-69.0) |
| Massachusetts | 52.2 (51.8-52.6) |
| Michigan | 81.7 (81.2-82.1) |
| Minnesota | 71.8 (71.2-72.4) |
| Mississippi | 101.9 (101.0-102.9) |
| Missouri | 79.3 (78.7-79.9) |
| Montana | 80.8 (79.4-82.2) |
| Nebraska | 91.0 (89.9-92.1) |
| Nevada | 73.4 (72.5-74.3) |
| New Hampshire | 76.1 (75.0-77.3) |
| New Jersey | 58.7 (58.3-59.1) |
| New Mexico | 68.6 (67.7-69.6) |
| New York | 64.2 (64.0-64.5) |
| North Carolina | 80.2 (79.7-80.7) |
| North Dakota | 84.7 (83.0-86.3) |
| Ohio | 100.1 (99.6-100.5) |
| Oklahoma | 124.8 (123.9-125.7) |
| Oregon | 80.9 (80.2-81.6) |
| Pennsylvania | 72.8 (72.4-73.1) |
| Rhode Island | 87.7 (86.4-89.1) |
| South Carolina | 80.9 (80.2-81.5) |
| South Dakota | 81.7 (80.2-83.2) |
| Tennessee | 95.4 (94.8-96.0) |
| Texas | 80.6 (80.3-80.9) |
| Utah | 41.8 (41.1-42.6) |
| Vermont | 102.5 (100.6-104.4) |
| Virginia | 60.6 (60.1-61.1) |
| Washington | 77.4 (76.9-78.0) |
| West Virginia | 131.9 (130.6-133.1) |
| Wisconsin | 69.0 (68.5-69.5) |
| Wyoming | 93.6 (91.4-95.7) |
| Overall | 75.4 (75.3-75.4) |
