# Supplementary material for: Mortality trends and disparities for coexisting chronic obstructive pulmonary disease and cardiovascular disease: A retrospective analysis of deaths in the United States from 1999–2020
Source: PLoS One. 2025 Feb 4;20(2):e0317592. doi: 10.1371/journal.pone.0317592 (PMC11793733; doi:10.1371/journal.pone.0317592)
Supplement: S8 Table — (DOCX) [file pone.0317592.s008.docx]

**S8 Table.** Cardiovascular Disease and Chronic Obstructive Pulmonary Disease related Age-Adjusted Mortality Rate per 100,000 Stratified by Census Region in Adults in the United States 1999-2020.

| Census Region | Year | Age-Adjusted Rate (95% CI) |
| --- | --- | --- |
| Northeast | 1999 | 77.0 (76.1-77.9) |
| Northeast | 2000 | 76.4 (75.5-77.3) |
| Northeast | 2001 | 74.5 (73.6-75.3) |
| Northeast | 2002 | 73.5 (72.6-74.4) |
| Northeast | 2003 | 71.4 (70.6-72.2) |
| Northeast | 2004 | 70.9 (70.1-71.8) |
| Northeast | 2005 | 71.4 (70.5-72.2) |
| Northeast | 2006 | 67.4 (66.6-68.2) |
| Northeast | 2007 | 66.2 (65.4-67.0) |
| Northeast | 2008 | 67.8 (67.1-68.6) |
| Northeast | 2009 | 64.4 (63.6-65.1) |
| Northeast | 2010 | 64.9 (64.1-65.6) |
| Northeast | 2011 | 64.9 (64.1-65.6) |
| Northeast | 2012 | 63.1 (62.3-63.8) |
| Northeast | 2013 | 62.1 (61.4-62.9) |
| Northeast | 2014 | 59.0 (58.3-59.8) |
| Northeast | 2015 | 60.8 (60.0-61.5) |
| Northeast | 2016 | 58.7 (58.0-59.4) |
| Northeast | 2017 | 59.1 (58.4-59.8) |
| Northeast | 2018 | 58.3 (57.6-59.0) |
| Northeast | 2019 | 57.4 (56.7-58.0) |
| Northeast | 2020 | 64.7 (64.0-65.4) |
| Northeast | **Overall** | 65.7 (65.5-65.8) |
| Midwest | 1999 | 84.6 (83.7-85.5) |
| Midwest | 2000 | 81.9 (81.1-82.8) |
| Midwest | 2001 | 82.6 (81.8-83.5) |
| Midwest | 2002 | 82.5 (81.7-83.4) |
| Midwest | 2003 | 82.0 (81.2-82.8) |
| Midwest | 2004 | 79.6 (78.8-80.4) |
| Midwest | 2005 | 82.7 (81.9-83.5) |
| Midwest | 2006 | 79.5 (78.7-80.4) |
| Midwest | 2007 | 77.5 (76.7-78.3) |
| Midwest | 2008 | 81.5 (80.7-82.3) |
| Midwest | 2009 | 77.2 (76.4-78.0) |
| Midwest | 2010 | 77.5 (76.8-78.3) |
| Midwest | 2011 | 79.9 (79.1-80.7) |
| Midwest | 2012 | 78.3 (77.6-79.1) |
| Midwest | 2013 | 79.5 (78.7-80.2) |
| Midwest | 2014 | 77.2 (76.4-77.9) |
| Midwest | 2015 | 79.7 (78.9-80.5) |
| Midwest | 2016 | 78.0 (77.2-78.7) |
| Midwest | 2017 | 80.1 (79.3-80.9) |
| Midwest | 2018 | 79.3 (78.5-80.0) |
| Midwest | 2019 | 79.1 (78.3-79.8) |
| Midwest | 2020 | 90.6 (89.8-91.4) |
| Midwest | **Overall** | 80.5 (80.3-80.6) |
| South | 1999 | 81.4 (80.6-82.1) |
| South | 2000 | 80.4 (79.7-81.1) |
| South | 2001 | 79.8 (79.1-80.5) |
| South | 2002 | 80.6 (79.9-81.3) |
| South | 2003 | 80.8 (80.1-81.5) |
| South | 2004 | 76.5 (75.9-77.2) |
| South | 2005 | 80.1 (79.4-80.7) |
| South | 2006 | 76.4 (75.8-77.1) |
| South | 2007 | 76.0 (75.3-76.6) |
| South | 2008 | 76.7 (76.1-77.4) |
| South | 2009 | 75.7 (75.1-76.4) |
| South | 2010 | 76.0 (75.4-76.6) |
| South | 2011 | 75.2 (74.6-75.9) |
| South | 2012 | 75.7 (75.1-76.3) |
| South | 2013 | 76.5 (75.9-77.1) |
| South | 2014 | 73.9 (73.3-74.4) |
| South | 2015 | 75.9 (75.3-76.4) |
| South | 2016 | 77.2 (76.7-77.8) |
| South | 2017 | 79.3 (78.7-79.9) |
| South | 2018 | 79.5 (78.9-80.1) |
| South | 2019 | 79.2 (78.6-79.7) |
| South | 2020 | 88.8 (88.2-89.4) |
| South | **Overall** | 78.3 (78.2-78.4) |
| West | 1999 | 86.5 (85.5-87.5) |
| West | 2000 | 83.1 (82.2-84.1) |
| West | 2001 | 81.9 (81.0-82.8) |
| West | 2002 | 81.2 (80.3-82.2) |
| West | 2003 | 81.3 (80.4-82.2) |
| West | 2004 | 77.7 (76.8-78.6) |
| West | 2005 | 79.8 (78.9-80.6) |
| West | 2006 | 75.3 (74.5-76.2) |
| West | 2007 | 73.7 (72.9-74.6) |
| West | 2008 | 74.0 (73.2-74.9) |
| West | 2009 | 70.9 (70.1-71.7) |
| West | 2010 | 72.2 (71.5-73.0) |
| West | 2011 | 72.8 (72.0-73.6) |
| West | 2012 | 70.2 (69.5-71.0) |
| West | 2013 | 71.6 (70.9-72.4) |
| West | 2014 | 67.3 (66.6-68.1) |
| West | 2015 | 69.3 (68.6-70.0) |
| West | 2016 | 69.4 (68.6-70.1) |
| West | 2017 | 70.3 (69.6-71.0) |
| West | 2018 | 68.5 (67.9-69.2) |
| West | 2019 | 67.3 (66.6-67.9) |
| West | 2020 | 72.9 (72.2-73.6) |
| West | **Overall** | 73.7 (73.5-73.8) |
