# Supplementary material for: Mortality trends and disparities for coexisting chronic obstructive pulmonary disease and cardiovascular disease: A retrospective analysis of deaths in the United States from 1999–2020
Source: PLoS One. 2025 Feb 4;20(2):e0317592. doi: 10.1371/journal.pone.0317592 (PMC11793733; doi:10.1371/journal.pone.0317592)
Supplement: S9 Table — (DOCX) [file pone.0317592.s009.docx]

**S9 Table.** Overall Cardiovascular Disease and Chronic Obstructive Pulmonary Disease–related Age-Adjusted Mortality Rates per 100,000 in Adults in the Metropolitan and Non-metropolitan areas in the United States, 1999 to 2020

| Age-Adjusted Rate (95% CI) | | |
| --- | --- | --- |
| Year | **Metropolitan** | **Nonmetropolitan** |
| 1999 | 79.6 (79.1-80.1) | 93.6 (92.5-94.6) |
| 2000 | 77.6 (77.1-78.0) | 93.1 (92.0-94.1) |
| 2001 | 76.6 (76.2-77.1) | 93.5 (92.5-94.6) |
| 2002 | 76.2 (75.8-76.7) | 95.0 (94.0-96.1) |
| 2003 | 75.6 (75.2-76.0) | 95.5 (94.5-96.5) |
| 2004 | 72.9 (72.4-73.3) | 92.1 (91.1-93.1) |
| 2005 | 74.9 (74.5-75.3) | 96.5 (95.5-97.5) |
| 2006 | 71.4 (71.0-71.8) | 91.7 (90.8-92.7) |
| 2007 | 69.9 (69.5-70.3) | 91.9 (91.0-92.9) |
| 2008 | 71.3 (70.9-71.7) | 94.8 (93.8-95.8) |
| 2009 | 68.5 (68.1-68.9) | 93.0 (92.0-94.0) |
| 2010 | 69.1 (68.7-69.5) | 93.2 (92.2-94.1) |
| 2011 | 69.5 (69.1-69.9) | 93.8 (92.8-94.7) |
| 2012 | 68.0 (67.6-68.4) | 94.9 (93.9-95.8) |
| 2013 | 68.9 (68.6-69.3) | 94.7 (93.7-95.7) |
| 2014 | 65.8 (65.5-66.2) | 92.6 (91.7-93.6) |
| 2015 | 67.3 (66.9-67.7) | 97.6 (96.6-98.5) |
| 2016 | 67.1 (66.8-67.5) | 97.2 (96.2-98.1) |
| 2017 | 68.3 (67.9-68.6) | 100.6 (99.7-101.6) |
| 2018 | 67.5 (67.2-67.9) | 101.2 (100.2-102.1) |
| 2019 | 66.7 (66.3-67.0) | 101.9 (100.9-102.8) |
| 2020 | 74.8 (74.5-75.2) | 113.9 (112.9-114.9) |
| Overall | 70.9 (70.8-71.0) | 96.2 (96.0-96.5) |
