# Supplementary material for: Mortality trends and disparities for coexisting chronic obstructive pulmonary disease and cardiovascular disease: A retrospective analysis of deaths in the United States from 1999–2020
Source: PLoS One. 2025 Feb 4;20(2):e0317592. doi: 10.1371/journal.pone.0317592 (PMC11793733; doi:10.1371/journal.pone.0317592)
Supplement: S10 Table — (DOCX) [file pone.0317592.s010.docx]

**S10 Table.** Overall Cardiovascular Disease (CVD) Alone and Chronic Obstructive Pulmonary Disease (COPD) Alone–related Age-Adjusted Mortality Rates per 100,000 in the United States, 1999 to 2020

| Age-Adjusted Rate (95% CI) | | |
| --- | --- | --- |
| Year | **CVD Alone** | **COPD Alone** |
| 1999 | 798.5 (797.2-799.8) | 136.8 (136.3-137.4) |
| 2000 | 784.3 (783.0-785.6) | 133.8 (133.2-134.3) |
| 2001 | 766.8 (765.5-768.1) | 132.9 (132.3-133.4) |
| 2002 | 760.1 (758.8-761.3) | 132.8 (132.2-133.3) |
| 2003 | 742.5 (741.2-743.7) | 132.2 (131.7-132.7) |
| 2004 | 709.2 (708.0-710.4) | 127.6 (127.1-128.2) |
| 2005 | 702.4 (701.2-703.5) | 132.6 (132.1-133.1) |
| 2006 | 675.0 (673.9-676.2) | 126.2 (125.7-126.7) |
| 2007 | 655.8 (654.7-656.9) | 124.0 (123.5-124.5) |
| 2008 | 650.9 (649.8-652.0) | 127.2 (126.8-127.7) |
| 2009 | 625.2 (624.1-626.3) | 122.4 (121.9-122.9) |
| 2010 | 621.2 (620.1-622.2) | 122.6 (122.1-123.1) |
| 2011 | 611.6 (610.6-612.7) | 123.4 (122.9-123.8) |
| 2012 | 601.4 (600.4-602.5) | 121.1 (120.6-121.5) |
| 2013 | 602.1 (601.1-603.1) | 122.3 (121.9-122.8) |
| 2014 | 593.6 (592.6-594.6) | 117.7 (117.2-118.1) |
| 2015 | 601.4 (600.4-602.4) | 120.4 (119.9-120.8) |
| 2016 | 596.0 (595.0-597.0) | 119.0 (118.6-119.5) |
| 2017 | 602.2 (601.3-603.2) | 120.1 (119.6-120.5) |
| 2018 | 599.7 (598.8-600.7) | 118.1 (117.6-118.5) |
| 2019 | 595.6 (594.6-596.5) | 115.3 (114.9-115.8) |
| 2020 | 691.0 (690.0-692.0) | 125.4 (125.0-125.8) |
| Overall | 656.0 (655.8-656.2) | 124.5 (124.4-124.6) |
